# Associations between media use at bedtime and sleep: a cross-sectional analysis on differences between girls and boys

**DOI:** 10.3389/fpsyg.2024.1290935

**Published:** 2024-07-12

**Authors:** Tanja Poulain, Cornelia Hilbert, Annelie Grundmann, Wieland Kiess

**Affiliations:** ^1^Faculty of Medicine, LIFE Leipzig Research Center for Civilization Diseases, Leipzig University, Leipzig, Germany; ^2^Department of Women and Child Health, Hospital for Children and Adolescents and Center for Paediatric Research (CPL), Leipzig University, Leipzig, Germany

**Keywords:** sleep, screen time, media use, bedtime, sex differences

## Abstract

**Objectives:**

This study investigated associations between the use of electronic media and sleep in children and young adolescents, with a specific focus on the moderation of associations by sex and age.

**Methods:**

Between 2021 and 2022, 453 10- to 14-year-old children participating in the LIFE Child cohort study (Germany) reported on their use of electronic media (daily screen time, use at bedtime, device ownership) and on their sleep difficulties (Sleep Self Report). Associations between media use and sleep as well as interactions with age and sex were assessed using linear regression analyses.

**Results:**

The analyses revealed significant associations between the use of media at bedtime and bedtime problems (in girls only), sleep behavior problems (in girls only), and daytime sleepiness (in girls and boys). Daily screen time, in contrast, was associated with none of the sleep difficulties. The number of media devices owned by the child was only associated with bedtime problems in girls, and this association lost statistical significance once media use at bedtime was included as further predictor.

**Conclusion:**

The findings underline the potentially sleep-disturbing role of electronic media at bedtime. Furthermore, they suggest that this effect is more pronounced in girls than in boys.

## 1 Introduction

Sleep is essential for healthy development. Therefore, the observed increase in sleep difficulties (Kronholm et al., 2015) and the decrease in sleep durations (Keyes et al., 2015) in children and adolescents in the last decades are worrisome. One factor that might be related to sleep onset delay, shorter sleep durations, and daytime sleepiness is the use of electronic media, which has increased considerably in the last years, especially regarding mobile and internet-enabling devices (Auhuber et al., 2019; Rideout and Robb, 2019). Several studies reported associations between the use of electronic media and sleep in children (Lund et al., 2021; Zhang et al., 2021). Longitudinal studies provide evidence for both, effects of media use on sleep (Johnson et al., 2004; Nuutinen et al., 2013; Schweizer et al., 2017) and effects of sleep on media use (Barlett et al., 2012; Tavernier and Willoughby, 2014). Some longitudinal studies also suggest bidirectional effects (Magee et al., 2014; Poulain et al., 2019). On the one hand, high media use can disturb sleep by increasing general arousal, by the presence of blue light, and by delaying the process of falling asleep and, therefore, shortening the duration of sleep (LeBourgeois et al., 2017). On the other hand, sleep difficulties such as daytime sleepiness or difficulties falling asleep might tempt children to use electronic media, even in the evening or at night (Magee et al., 2014; Tavernier and Willoughby, 2014).

It is argued that media use at bedtime might be especially sleep-disturbing (Lund et al., 2021) and can be considered a general risk factor for unhealthy child development (Gentile et al., 2017). However, the majority of previous studies assessed only mere screen time (irrespectively of the time of the day), and the few studies assessing screen time at bedtime usually did not assess screen time over the day as a separate media use parameter. A study by Nathanson and Beyens assessed daily media use and media use at bedtime and found that both were associated with significantly poorer sleep outcomes in preschool children (Nathanson and Beyens, 2018). However, daily media use and bedtime media use were not included in the same statistical model, therefore, it is not possible to tell whether the associations exist independently of each other. The present study investigated associations between media use and sleep in a large sample of young adolescents. Importantly, we distinguished different parameters of media use—screen time (irrespectively whether before going to bed or at other times during the day), number of devices owned by the child (as an indicator of how many devices might be used), and media use at bedtime (which is supposed to be especially sleep-disturbing)—and investigated whether associations with sleep are independent. Based on previous study findings, we expected all of the assessed media use parameters to be associated with more sleep difficulties. The strongest associations, however, were expected for media use at bedtime.

Another aim of this study was to assess whether associations between media use and sleep are moderated by child age and sex. Only a few previous studies assessed the question of moderation (Lund et al., 2021). One study showed adverse effects of stress linked to social media use on later sleep latency and daytime sleepiness in girls but not in boys (van der Schuur et al., 2019). In another study, in contrast, electronic media in the bedroom were associated with poorer sleep habits in boys but not in girls (Nuutinen et al., 2013). Regarding age, findings of a systematic review suggest that associations between electronic media use and sleep difficulties are stronger in 6- to 15-year-olds (i.e., in children using electronic media more and more independently) than in 0- to 5-year-olds (i.e., in children whose media use in mainly guided by their parents) (Lund et al., 2021). However, this suggestion was based on comparisons between different studies/samples. We are not aware of any studies reporting age differences within the same sample, e.g., a sample including younger and older school-aged children.

Based on the previous study findings, we expected screen time and media use at bedtime to show stronger associations with sleep in girls than boys, while the number of media devices owned by the child should show stronger associations with sleep in boys than girls. As our study sample included only children within a rather small age range, we expected no moderation of any media-sleep association by child age.

## 2 Materials and methods

### 2.1 Participants

The study was realized within the LIFE Child study, a longitudinal cohort study conducted at the Leipzig Research Center for Civilization Diseases at Leipzig University (Quante et al., 2012; Poulain et al., 2017). The study aims to assess healthy child development from the prenatal period to early adulthood and to investigate the development of non-communicable diseases, e.g., obesity and depression. Child participants are recruited until the age of 16 years via advertisement at different health institutions. All participants are invited to attend follow-up visits taking place once a year.

Data for the present study were collected between 2021 and 2022, i.e., during the COVID-19 pandemic. This period was characterized by restrictions on everyday life and contact. However, schools were open. All children having completed the questionnaires on media use and sleep were eligible for analysis. This condition was met by 453 children aged 10–14 years (229 boys, 224 girls, mean age 12.19 years). In 146 cases, the same children had completed the questionnaires at more than one time point. Therefore, all analyses were adjusted for multiple visits of the same child.

The LIFE Child study was designed in accordance with the Declaration of Helsinki and the study protocol was approved by the Ethics Committee of the Medical Faculty of the Leipzig University (Reg. No. 477/19-ek). The parents of all participating children provided informed written consent before the inclusion of their children in the study.

### 2.2 Measurements

Media use of participants was assessed using a self-developed media use questionnaire completed by the children themselves. For the present study, three questions were analyzed. The first question—“Do you usually use screen-based media (for at least 15 min) in the hour before going to bed?” (response options: no, yes)—was analyzed to assess media use at bedtime. The second question—“Which of the following media devices do you own (personally/in your bedroom)? TV—laptop/tablet—personal computer—smartphone—smartwatch or something similar—games console” (response options for each device: no, yes)—was used to assess the number of media devices owned by the child. For further analysis, the number of owned devices was summed up. The third question—“On a normal weekday, how much time do you spend using screen-based media?” (response options: never, 30 min, 1 h, 1.5 h, […] 12 h)—was analyzed to assess screen time. For further analysis, responses were transformed to hours (0, 0.5, 1, 1.5 […] 12).

Participants’ sleep difficulties were assessed using the Sleep Self Report (Owens et al., 2000; Schwerdtle et al., 2010), completed by children themselves. This screening questionnaire consists of 18 items measuring three domains of sleep difficulties: bedtime problems (9 items), sleep behavior problems (6 items), and daytime sleepiness (3 items). The items are presented in Table 1. Response options for each item were “usually (5–7 times/week”), “sometimes (2–4 times/week),” and “rarely (0–1 time/week) or never.” For each domain, the values (1, 2, or 3) of the single items were summed up to a sum score, where higher scores indicate more sleep difficulties.

**Table 1:** Items of the sleep self report.

Domain	Items
Bedtime problems	Do you go to bed at the same time every night on school nights? (R) Do you fall asleep in the same bed every night? (R) Do you fall asleep in about 20 min? (R) Do you fight with your parents about going to bed? Is it hard for you to go to bed? Are you ready for bed at your usual bedtime? (R) Are you afraid of the dark? Are you afraid of sleeping alone? Do you stay up late when your parents think you are asleep?
Sleep behavior problems	Do you think you sleep too little? Do you think you sleep too much? Do you wake up at night when your parents think you are asleep? Do you have trouble falling back to sleep if you wake up during the night? Do you have nightmares? Do you sometimes go to someone’s bed during the night?
Daytime sleepiness	Do you have trouble waking up in the morning? Do you feel sleepy during the day? Do you feel rested after a night’s sleep? (R)

Response options: 1: rarely (0–1/week or never), 2: sometimes (2–4 times/week), 3: usually (5–7/week).

(R): Reverse scoring.

Age (in years), sex (male, female) and socioeconomic status (SES, indicated by the family’s net income) were included as covariates and—in the case of age and sex—moderators. For the assessment of income, parents were asked to indicate their monthly net income. They could choose between 14 response categories ranging from “less than 500 Euro” to “10.000 Euro or more.” The responses were combined to a score ranging from 1 (lowest income) to 7 (highest income), as described in Lampert et al. (2014).

### 2.3 Statistical analysis

All analyses were performed using R (R Core Team, 2019). Characteristics of the study sample were presented as means and standard deviations (for continuous variables) and as counts and percentages (for categorical variables). Sex differences in the sociodemographic, media use, and sleep variables were assessed using t-tests (for continuous variables) and Chi-squared tests (for dichotomous variables).

Associations between media use parameters (included as independent variables) and sleep parameters (included as dependent variables) were assessed using hierarchical linear models, with the ID of each participant included as random factor (package lme4). All associations were adjusted for the covariates. Statistical significance was indicated by a *p*-value < 0.05.

In the moderator analyses, we checked for interactions between each independent variable and sex and age. A significant interaction indicated a moderation of the association by the corresponding variable. Non-significant interactions were not presented.

## 3 Results

### 3.1 Characteristics of the study sample

The analyzed sample comprised 453 children aged 10–14 years. Characteristics of the whole sample and of girls and boys, separately, are presented in Table 2. As can be seen, the income of the participating families was rather high, indicating an SES above average.

**Table 2:** Characteristics of the study sample (*n* = 453).

		Possible range	All children *n* = 453	Girls *n* = 224 (49%)	Boys *n* = 229 (51%)	Significance of sex differences
Age	Mean (sd)	10–14	12.19 (0.88)	12.22 (0.84)	12.17 (0.92)	
Income	Mean (sd)	1–7	5.51 (1.44)	5.54 (1.43)	5.49 (1.45)	
Media use at bedtime	Yes (*n*/%)		259 (57%)	130 (58%)	129 (56%)	
Number of media devices	Mean (sd)	0–6	2.65 (1.30)	2.33 (1.19)	2.96 (1.34)	***
Screen time (minutes/day)	Mean (sd)	0–720	139.12 (94.13)	137.19 (98.14)	141.00 (90.21)	
Bedtime problems	Mean (sd)	9–27	13.09 (2.73)	13.48 (2.76)	12.71 (2.65)	**
Sleep behavior problems	Mean (sd)	6–18	8.27 (2.16)	8.42 (1.96)	8.11 (2.33)	
Daytime sleepiness	Mean (sd)	3–9	5.49 (1.43)	5.55 (1.45)	5.42 (1.41)	

Regarding media use, more than half of the participants (57%) used screen-based media in the hour before going to bed. On average, boys owned significantly more media devices (mean = 2.96) than girls (mean = 2.33). Media use at bedtime and overall screen time, in contrast, did not differ significantly between girls and boys.

With respect to sleep, the average bedtime problems score was significantly higher in girls (13.48) than in boys (12.71), indicating more problems at bedtime in girls than boys. In the other sleep domains, no significant sex differences were observed.

### 3.2 Associations between media use and sleep parameters

**Figure 1:**
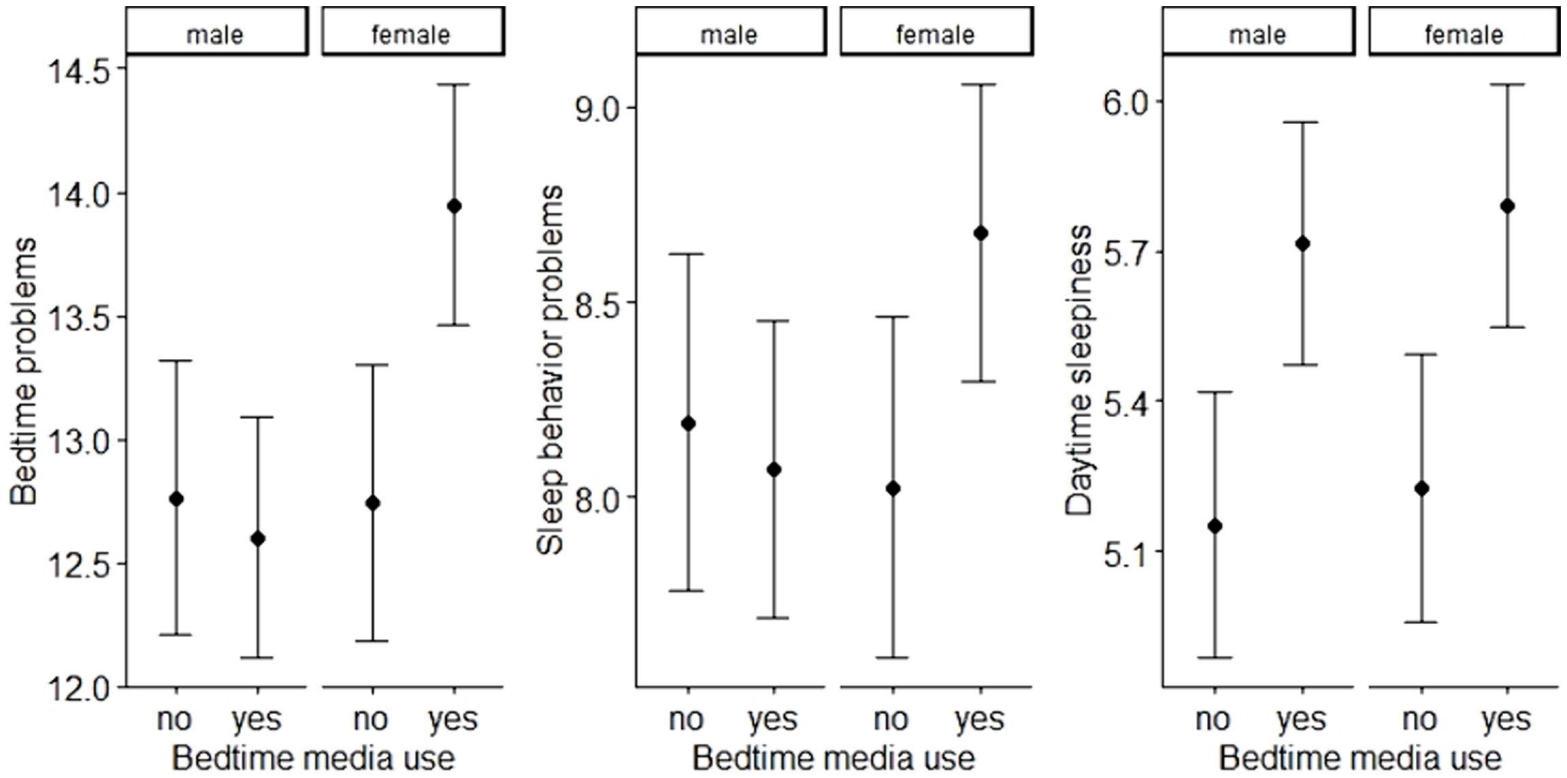
Associations between bedtime media use and bedtime problems (left), sleep behavior problems (middle), and daytime sleepiness (right) in boys and girls; the associations between bedtime media use and bedtime problems as well as sleep behavior problems were significant in girls only.

The analyses revealed significant associations between bedtime media use and sleep difficulties in all three domains (bedtime problems, sleep behavior problems, daytime sleepiness, see Table 3). Interestingly, the associations between bedtime media use and bedtime problems and sleep behavior problems were only significant in girls (*b* = 1.20 and 0.65, *p* < 0.001 and 0.010), but not in boys (*b* = −0.16 and −0.12, *p* = 0.615 and 0.635). Figure 1 illustrates the associations between media use at bedtime and sleep difficulties in the three domains, separated by child sex.

**Table 3:** Associations between sleep difficulties (as dependent variable) and the media use parameters.

Dependent variable	Independent variable	Overall effect	Effect in girls	Effect in boys
**Media use at bedtime**			
Bedtime problems	(b + 95% CI)		1.20 (0.57 – 1.84)***	−0.16 (−0.79 – 0.47)
	*p*		<0.001	0.615
Sleep behavior problems	(b + 95% CI)		0.65 (0.16 – 1.14)*	−0.12 (−0.60 – 0.37)
	*p*		0.010	0.635
Daytime sleepiness	(b + 95% CI)	0.56 (0.30 – 0.83)***		
	*p*	<0.001		
**Number of media devices owned by the child**			
Bedtime problems	(b + 95% CI)		0.47 (0.20 – 0.75)**	−0.02 (−0.26 – 0.21)
	*p*		0.001	0.844
Sleep behavior problems	(b + 95% CI)	0.03 (−0.10 – 0.17)		
	*p*	0.625		
Daytime sleepiness	(b + 95% CI)	0.03 (−0.07 – 0.14)		
	*p*	0.541		
**Daily screen time**			
Bedtime problems	(b + 95% CI)	0.06 (−0.09 – 0.20)		
	*p*	0.453		
Sleep behavior problems	(b + 95% CI)	0.08 (−0.03 – 0.20)		
	*p*	0.148		
Daytime sleepiness	(b + 95% CI)	0.06 (−0.03 – 0.14)		
	*p*	0.187		

With respect to the number of media devices owned by the child, we observed a significant association with bedtime problems, which, similar as for media use at bedtime, was only significant in girls (*b* = 0.47, *p* = 0.001) but not in boys (*b* = −0.02, *p* = 0.844). In a model with media use at bedtime and the number of media devices included simultaneously as independent variables, only the association between media use at bedtime and bedtime problems remained significant (*b* = 0.50, *p* = 0.033), while the association between the number of media devices and bedtime problems lost statistical significance (*b* = 0.12, *p* = 0.178). The number of media devices was not significantly associated with sleep behavior problems or daytime sleepiness (see Table 3). Finally, the analyses revealed no significant associations between daily screen time and any of the sleep difficulties (see Table 3).

Summarizing, we observed significant associations between media use at bedtime and sleep disturbances. With the exception of the association with daytime sleepiness, these associations were only significant in girls. All associations—for the whole sample (when the association was not moderated by sex) or separated by sex (when the association was moderated by sex)—are presented in Table 3. Age did not moderate any of the associations between sleep parameters and media use parameters.

## 4 Discussion

This study investigated the association between different media use parameters and sleep difficulties in a sample of German children and young adolescents. The mean scores reflecting difficulties in the different sleep domains (bedtime problems, sleep behavior problems, daytime sleepiness) were slightly higher than the values reported in a representative German sample in 2010 (Schwerdtle et al., 2010) and a representative Dutch sample in 2019 (Steur et al., 2019), indicating higher amounts of sleep difficulties in our sample of children and adolescents. This observation is in line with previous findings in the LIFE Child study and might be explained by differences regarding the time point of assessment (2010 versus 2021/2022), the age of the included children (including versus excluding children younger than 10 years), and the environment in which children are growing up (rural and urban versus urban only) (Poulain et al., 2019; Lewien et al., 2021). Furthermore, it is also important to note that our study was conducted during the COVID-19 pandemic. During this time, sleep disorders were particularly common in school-age children (Sharma et al., 2021). Regarding the media use of children in the present sample, the results emphasize the omnipresence of electronic devices in the everyday lives of children and young people, even at bedtime. Again, the COVID-19 pandemic might be one reason for this (Serra et al., 2021).

### 4.1 Associations between the use of electronic media and sleep

The analyses revealed several significant associations between the different media use parameters and sleep. The first main finding is that media use at bedtime showed the strongest associations with sleep parameters. The number of media devices owned by the child, in contrast, showed only an association with bedtime problems; and this association lost statistical significance once media use at bedtime was included as a further predictor. Mere screen time was associated with none of the assessed sleep difficulties. These findings suggest that media use at bedtime might be especially sleep disturbing. Associations between sleep difficulties and the number of media devices owned by children or screen time, in contrast, may actually be explained by an underlying association between sleep difficulties and media use at bedtime.

In general, the use of electronic media, especially of interactive media (Bartel et al., 2015) such as gaming or social networking, may increase arousal, e.g., because one has received a specific message or is enthusiastic about a video game. This arousal might be especially harmful at bedtime as it delays the process of falling asleep. Not being able to finish a video game with other online players or missing/not being able to read or react to the newest messages on social media platforms because one has to go to sleep can cause additional stress [fear of missing out (Gupta and Sharma, 2021; Li et al., 2021)]. This stress can further interfere with the process of falling asleep and might have an impact on night sleep and daytime sleepiness (Scott and Woods, 2018). In addition, blue light at bedtime, as diffused by screens, has been shown to be related to lower sleep quality in adolescents and young adults (Rafique et al., 2020). Therefore, blue light might be another explanation for the observed associations between media use at bedtime and sleep difficulties. However, it is important to consider that the data assessed in the present study were cross-sectional. Therefore, it is not possible to make assumptions on the direction of effects. As suggested so far, media use at bedtime might cause sleep difficulties. At the same time, however, sleep difficulties might also cause media use. For example, difficulties falling asleep might motivate children to use electronic media at bedtime (Magee et al., 2014; Tavernier and Willoughby, 2014).

The second main finding of the present study is that, with the exception of the association between media use at bedtime and daytime sleepiness, the associations between media use (at bedtime) and sleep difficulties were only significant in girls but not in boys. One possible reason for this finding is that the arousal or stress induced by the late use of electronic media is higher in girls than in boys, e.g., because girls use more social media than boys, a phenomenon shown in previous research (Twenge and Martin, 2020). Another possible reason for the finding is that the arousal or stress induced by the late use of electronic media has a stronger sleep-disturbing effect in girls than in boys. This explanation is supported by findings of a study showing associations between stress induced by social media and sleep latency and daytime sleepiness in girls but not in boys (van der Schuur et al., 2019).

In contrast to sex, age did not moderate the associations between the use of electronic media and sleep difficulties in children. This finding indicates that the observed associations between media use (at bedtime) and sleep difficulties were comparably strong in children aged 10 years and in young adolescents aged 14 years, suggesting that the associations are hardly influenced by puberty-related hormonal or behavioral changes. However, it is important to note that the age range in our study was rather small and did not include younger children. As suggested by a recent review (Lund et al., 2021), associations between electronic media use and sleep difficulties might be weaker in younger children, i.e., children whose media use is still monitored by their parents.

### 4.2 Strengths, limitations and future directions

Strengths of the study are the large sample size, the distinction between screen time, media use at bedtime, and the number of media devices owned by the child, and the consideration of sex and age as moderators in the association between media use and sleep difficulties. Due to a very limited number of children attending follow-up visits since 2021, we were only able to perform cross-sectional analyses. Therefore, assumptions about causal relationships remain speculative. Another limitation is that we did not investigate which kinds of media activities children engaged in at bedtime, e.g., social media, gaming, or watching videos. We also did not assess the use of non-screen-based media (e.g., reading). The associations with sleep disturbances might differ depending on the media activity. Also, the observed sex differences regarding the association between media use and sleep difficulties might actually be explained by sex differences in the engagement of different media activities. Another limitation is that the study was conducted during the COVID-19 pandemic. Even if schools were open, other everyday restrictions may have changed the sleep and media usage behavior of the participating children. Finally, the sample was not representative in terms of SES/income. Children from families with low SES/income were underrepresented.

Future studies might assess whether media use and sleep difficulties receded after the pandemic and whether associations between the two were comparable to those shown in the present study. They might also assess whether the use of non-screen-based media (at bedtime) show similar or different associations with sleep.

## 5 Conclusion

The findings of the present study show that the use of media at bedtime, but not the whole amount of screen time per day or the number of media devices in the bedroom, is associated with sleep difficulties in children and young adolescents, especially in girls. Due to the cross-sectional design, the results do not allow any assumptions about causality. Children who use electronic media at bedtime may have problems falling asleep, and children with sleep difficulties may use electronic media as a coping strategy. In both cases, however, it is recommended to limit the use of electronic media at bedtime, as staring at a lit screen is not a good strategy for falling asleep.

## Data availability statement

The datasets presented in this article are not readily available due to ethical restrictions. The LIFE Child study is a study collecting potentially sensitive information. Publishing data sets is not covered by the informed consent provided by the study participants. Furthermore, the data protection concept of LIFE requests that all (external as well as internal) researchers interested in accessing data sign a project agreement. Researchers that are interested in accessing and analyzing data collected in the LIFE Child study may contact the data use and access committee (forschungsdaten@medizin.uni-leipzig.de). Requests to access the datasets should be directed to forschungsdaten@medizin.uni-leipzig.de.

## Ethics statement

The studies involving humans were approved by Ethikkommission der Medizinischen Fakultät der Universität Leipzig, Stephanstraße 9A.1, 04103 Leipzig, Germany. The studies were conducted in accordance with the local legislation and institutional requirements. Written informed consent for participation in this study was provided by the participants’ legal guardians/next of kin.

## Author contributions

TP: Conceptualization, Formal analysis, Methodology, Writing – original draft. CH: Investigation, Writing – review & editing. AG: Investigation, Writing – review & editing. WK: Conceptualization, Supervision, Writing – review & editing.
